# Effects of the COVID-19 Lockdown on Air Pollutant Levels and Associated Reductions in Ischemic Stroke Incidence in Shandong Province, China

**DOI:** 10.3389/fpubh.2022.876615

**Published:** 2022-05-27

**Authors:** Han Wu, Zilong Lu, Jing Wei, Bingyin Zhang, Xue Liu, Min Zhao, Wenhui Liu, Xiaolei Guo, Bo Xi

**Affiliations:** ^1^Department of Epidemiology, School of Public Health, Qilu Hospital, Cheeloo College of Medicine, Shandong University, Jinan, China; ^2^Shandong Center for Disease Control and Prevention, and Academy of Preventive Medicine, Shandong University, Jinan, China; ^3^Department of Atmospheric and Oceanic Science, Earth System Science Interdisciplinary Center, University of Maryland, College Park, MD, United States; ^4^Department of Nutrition and Food Hygiene, School of Public Health, Cheeloo College of Medicine, Shandong University, Jinan, China; ^5^Information and Data Analysis Lab, School of Public Health, Cheeloo College of Medicine, Shandong University, Jinan, China

**Keywords:** lockdown, air pollution, ischemic stroke, COVID-19, incidence

## Abstract

**Background:**

Local governments in China took restrictive measures after the outbreak of COVID-19 to control its spread, which unintentionally resulted in reduced anthropogenic emission sources of air pollutants. In this study, we intended to examine the effects of the COVID-19 lockdown policy on the concentration levels of particulate matter with aerodynamic diameters of ≤1 μm (PM_1_), ≤2.5 μm (PM_2.5_), and ≤10 μm (PM_10_), nitrogen dioxide (NO_2_), sulfur dioxide (SO_2_), ozone (O_3_), and carbon monoxide (CO) and the potential subsequent reductions in the incidence of ischemic and hemorrhagic stroke in Shandong Province, China.

**Methods:**

A difference-in-difference model combining the daily incidence data for ischemic and hemorrhagic stroke and air pollutant data in 126 counties was used to estimate the effect of the COVID-19 lockdown on the air pollutant levels and ischemic and hemorrhagic stroke incident counts. The avoided ischemic stroke cases related to the changes in air pollutant exposure levels were further estimated using concentration-response functions from previous studies.

**Results:**

The PM_1_, PM_2.5_, PM_10_, NO_2_, and CO levels significantly decreased by −30.2, −20.9, −13.5, −46.3, and −13.1%, respectively. The O_3_ level increased by 11.5% during the lockdown compared with that in the counterfactual lockdown phase of the past 2 years. There was a significant reduction in population-weighted ischemic stroke cases (−15,315, 95% confidence interval [*CI*]: −27,689, −2,942), representing a reduction of 27.6% (95% *CI*: −49.9%, −5.3%). The change in the number of hemorrhagic stroke cases was not statistically significant. The total avoided PM_1_-, PM_2.5_-, PM_10_-, NO_2_-, and CO–related ischemic stroke cases were 739 (95% *CI*: 641, 833), 509 (95% *CI*: 440, 575), 355 (95% *CI*: 304, 405), 1,132 (95% *CI*: 1,024, 1,240), and 289 (95% *CI*: 236, 340), respectively.

**Conclusion:**

The COVID-19 lockdown indirectly reduced the concentration levels of PM_1_, PM_2.5_, PM_10_, NO_2_, and CO and subsequently reduced the associated ischemic stroke incidence. The health benefits due to the lockdown are temporary, and long-term measures should be implemented to increase air quality and related health benefits in the post-COVID-19 period.

## Introduction

COVID-19 broke out in China in December 2019. Within a few months, this disease spread around the world, becoming a global pandemic (1). The governments of most countries adopted lockdowns to retard the spread of COVID-19 (2). Chinese provincial governments took the Level I major public health emergency response action on 24 January 2020, and all cities implemented lockdowns. Thus, many industrial and human activities were restricted only to the essentials or a bare minimum (3–6).

Although the cost of implementing nationwide restrictions is undoubtedly enormous, the lockdowns contributed markedly to successfully controlling the spread of the pandemic. Moreover, they generated a range of unintended environmental and health benefits (7). Extensive studies have been conducted to evaluate the impacts of the COVID-19 lockdowns on air quality, as there were substantial reductions in the industrial and vehicle emissions of air pollutants due to the reduced anthropogenic sources (8–10). Most studies suggest that the lockdowns had positive effects on the improvement of regional air quality, especially in areas with severe air pollution (e.g., the Middle East, India, and China) (4, 9–12). Furthermore, some studies focused on the health benefits attributable to the COVID-19 lockdowns (13–18). For example, significant reductions in mortality and hospitalizations for cardiovascular diseases, such as atrial fibrillation, acute coronary syndrome, myocardial infarction, and ischemic stroke, were observed in both developed and developing countries (13–18).

In China, there were approximately 29 million prevalent stroke cases in 2019, and stroke has become the leading cause of death and disability-adjusted life-years (19). Accumulating evidence suggests that short-term exposure to air pollutants is associated with increased risks of stroke incidence and mortality (20). Therefore, the lowered air pollution levels due to the lockdowns may have reduced stroke-associated events. Several studies confirmed the causal effects of the COVID-19 lockdown on improved air quality and its subsequent health benefits. However, most of them focused on stroke mortality, and using mortality as the outcome of interest may underestimate the number of people affected by the lockdowns (3, 5, 17). In contrast, using stroke incidence as an outcome may greatly outnumber mortality events, and the estimated number of stroke incidence cases avoided because of the reduced air pollution levels due to the lockdowns may be greater than that of mortality, thus providing greater statistical power for examining the health benefits generated by the COVID-19 lockdowns.

To the best of our knowledge, no study has yet examined the reduction effect of the COVID-19 lockdowns on ischemic and hemorrhagic stroke incidence in China to date. In addition, most previous studies focused on the changes in commonly monitored air pollutants (i.e., PM_2.5_, PM_10_, SO_2_, NO_2_, O_3_, and CO), and only limited studies (from Croatia, France, India, Italy, and the western Mediterranean) examined the effect of the COVID-19 lockdowns on the PM_1_ level and relevant literature is scarce in China (21–25). In this study, we investigated the impacts of a COVID-19 lockdown on the concentrations of PM_1_ and other air pollutants (PM_2.5_, PM_10_, SO_2_, NO_2_, O_3_, and CO) and the potential subsequent reductions in ischemic and hemorrhagic stroke incidence in 126 counties in Shandong Province, China. We collected stroke incidence data from a stroke registry system, which covered all stroke case records from every medical institution (e.g., private clinics, community health service centers, and public hospitals) in the included counties.

## Materials and Methods

### Stroke Registry Data

Data on the county-specific daily incidence of stroke were obtained from the stroke registry system operated by the Shandong Center for Disease Control and Prevention (CDC). Patient admissions to all medical institutions with a stroke diagnosis in each county must be reported to this registry system. From 2017 to 2020, the system covered 126 of the 136 counties in Shandong, and the total population of these counties was 91 million, equivalent to 6.4% of the whole population of China.

In this stroke registry system, registry certificates were filled in by the attending physician who diagnoses patients according to their symptoms, inquiries, complaints, and medical inspection results. The diagnosis was then categorized according to the International Classification of Diseases version 10 (ICD-10). In addition, each patient was asked to report when clinical symptoms occurred, which was then recorded as the incidence date. Then, the registry certificates were reported to the registry system in real-time. The information on each certificate was validated by professionals in the CDC of each county within 7 days, who also checked for completeness and coding. In this study, we focused on ischemic stroke (ICD-10 code: I63) and hemorrhagic stroke (ICD-10 codes: I60-I61). Ethical approval was obtained from the Ethics Review Committee of Public Health, Shandong University (No. LL20211203).

### Air Pollution Data

Daily ambient PM_1_, PM_2.5_, PM_10_, NO_2_, SO_2_, O_3_, and CO data were collected from ChinaHighAirPollutants (CHAP, available at https://weijing-rs.github.io/product.html) datasets for Shandong province from 2017 to 2020 at a spatial resolution of 0.1° (≈10 km). These data were estimated using a developed space-time extremely randomized trees model, which integrates satellite remote sensing products, atmospheric reanalysis, and ground-based measurements to accomplish model simulations (26–32). The data on the predicted air pollutants levels were compared validly with ground-level measurements. The cross-validation coefficients of determination (CV-R^2^) were 0.82, 0.91, 0.86, 0.84, 0.84, 0.87, and 0.80, and the root-mean-square errors (RMSEs) were 10.86, 12.67, 24.34, 7.99, 10.07, 17.10, and 0.29 mg/m^3^ for daily concentrations of PM_1_, PM_2.5_, PM_10_, NO_2_, SO_2_, O_3_, and CO, respectively. The CHAP datasets have been widely applied in recent epidemiological studies evaluating the impact of exposure to ambient air pollutants on population health in China (33–35). For our analysis, the daily mean concentrations of PM_1_, PM_2.5_, PM_10_, NO_2_, SO_2_, O_3_, and CO for each county were estimated by calculating the average of the pixel values weighted by the proportion of the area of the county covered by the pixel, which enhanced the spatial representativeness of air pollution for each county.

### Statistical Analysis

**Figure 1:**
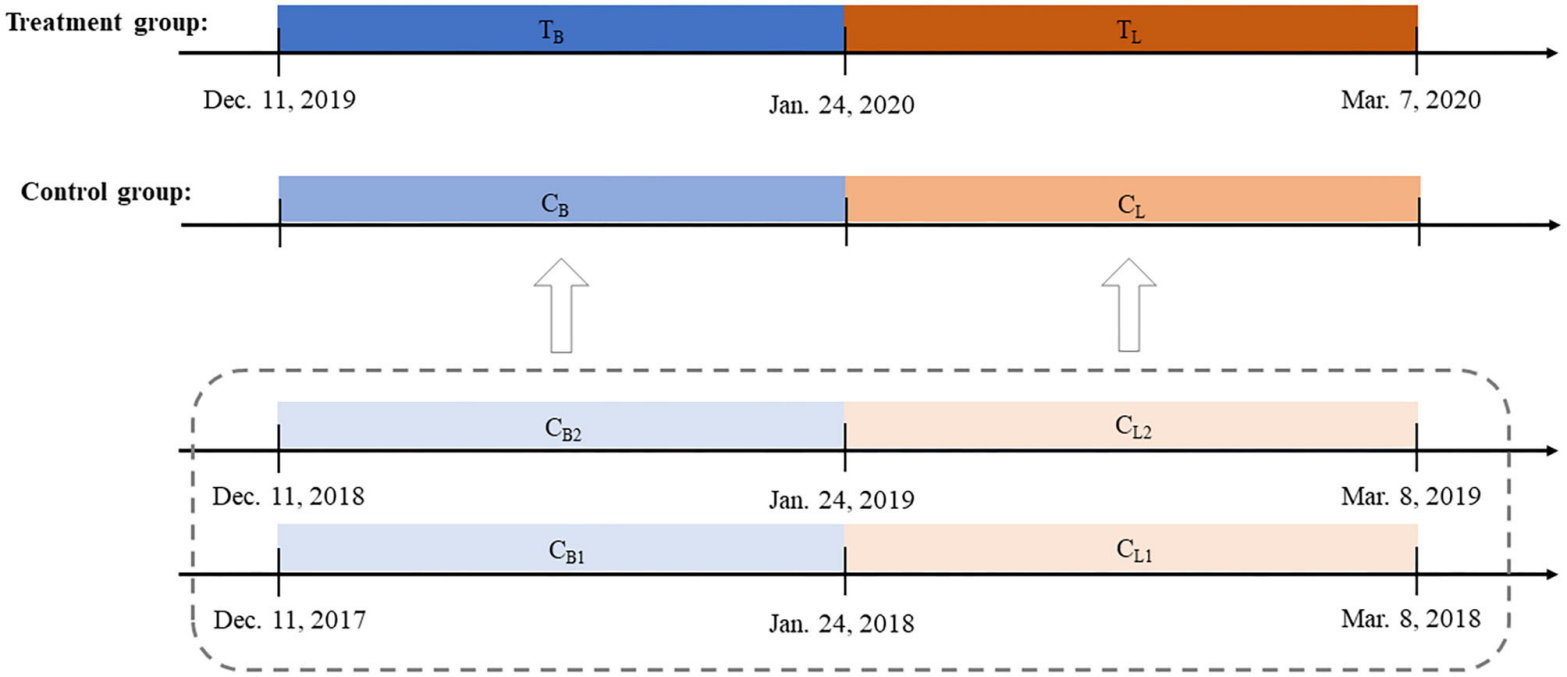
Settings of the difference-in-difference (DID) model.

In this study, we first calculated the changes in the levels of each air pollutant and the ischemic and hemorrhagic stroke counts. Then, we calculated the estimated avoided stroke incident cases attributable to these air pollution changes by county. We employed a quasi-experiment design and used a difference-in-difference (DID) approach to estimate the effect of the COVID-19 lockdown on air pollutant levels (PM_1_, PM_2.5_, PM_10_, SO_2_, NO_2_, O_3_, and CO) and ischemic and hemorrhagic stroke incident counts. Using this approach, we compared the same period in the past 2 years before the COVID-19 lockdown with a certain period in the year of the lockdown. Then, the net treatment effect of the lockdown on the air pollutant levels and stroke incident counts can be estimated. The detailed procedure is illustrated in Figure 1.

The government of Shandong province announced the Level I major public health emergency response on 24 January and lifted the Level I response on 7 March. In this context, the lockdown phase was defined as the period between 24 January and 7 March, which lasted for a sum of 44 days. We set the period between 11 December 2019, and 23 January 2020, as the baseline phase to make the time length of the baseline phase equal to that of the lockdown phase. Then, we created a DID model to quantify the change level of each air pollutant for each county. Taking PM_1_ as an example, we first selected four corresponding phases (C_B1_, C_L1_, C_B2_, and C_L2_) from 2017 to 2019, with C_B1_ denoting the mean daily PM_1_ concentration level from 11 December 2017 to 23 January 2018, C_L1_ the mean daily PM_1_ concentration level from 24 January 2018 to 8 March 2018, C_B2_ the mean daily PM_1_ concentration level from 11 December 2018 to 23 January 2019, and C_L2_ the mean daily PM_1_ concentration level from 24 January 2019 to 8 March 2019. Next, the means of C_B1_ and C_B2_ (i.e., C_B_) and C_L1_ and C_L2_ (i.e., C_L_) were calculated to form a PM_1_ concentration at the baseline and lockdown phases, respectively, as the counterfactual control groups. Then, we calculated the difference in the PM_1_ concentration levels of the treatment group and the control group during the lockdown phase (T_L_ – C_L_) and the difference between the two groups during the baseline phase (T_B_ – C_B_). The changes in the PM_1_ concentration level for each county related to the lockdown policy beyond background trends can be estimated using the above two differences: (T_L_ – C_L_) – (T_B_ – C_B_).

Three key assumptions of DID should be satisfied (36). First, the event shock should be completely exogenous. In this study, the COVID-19 lockdown in Shandong province is used as a quasi-experiment, and no one could have predicted the COVID-19 outbreak or the unprecedented nationwide lockdown in late 2019, which can be regarded as a “black swan.” Thus, the DID model used in this study meets the exogeneity assumption. Second, the exogenous shock should affect only the treatment units and not the potential control units. In this study, the 44 days before and after the day of the announcement of the lockdown policy (24 January 2020) were selected as the treatment group, and the same period in the past 2 years was selected as the control group. The COVID-19 outbreak in 2020 cannot affect the air quality levels in 2018 or 2019. Third, the outcome of interest between the treatment and control groups must have similar fits before the exogenous event occurs, otherwise known as the parallel trend assumption. If this assumption is satisfied, we can reasonably assume that the parallel trends of air pollutant concentrations over time would be the same for both groups. This assumption was examined using a regression model, which included the interaction term between time and the baseline period (18). We found that the coefficient for the interaction term is not statistically significant for each of the outcomes, indicating the parallel trend assumption for the DID model is plausible (Supplementary Table 1) (18).

Our preliminary analyses showed a statistically significant change in ischemic stroke cases. Thus, the following formula was used to estimate the counts of the avoided ischemic stroke cases related to changes in the air pollutant exposure levels, which was adopted from recent studies evaluating the impact of ambient air pollutants exposure on population health (3, 37, 38):


$$\begin{array}{l} \Delta C_{m}=B_{m}\times \left(e^{\beta \times \Delta ap_{m}}-1\right) \end{array}$$

Δ*C*_*m*_ indicates the avoided ischemic stroke incident cases for county *m, B*_*m*_ is the baseline counts (i.e., the average of the counts from 24 January to 7 March in 2018 and 2019) for ischemic stroke for county *m*, β represents the exposure-response effect estimates extracted from two previous studies (Supplementary Table 2) (39, 40), and Δ*ap*_*m*_ is the change in one specific air pollutant for county *m*.

All the above analyses were weighted by the population in each county. We conducted a sensitivity analysis by estimating the change of the sex- and age-adjusted incidence rates of ischemic stroke and hemorrhagic stroke during the lockdown phase in the DID model to further check the robustness of using the incident count of stroke as the outcome. The sex- and age-adjusted incidence rates were calculated using the population data for each county, which were collected from China's 2010 census survey.

The statistical analysis was carried out using R 4.0.3 (The R Project for Statistical Computing, Vienna, Austria), and two-sided values of *p* < 0.05 were considered statistically significant.

## Result

During the lockdown phase in 2020, the mean [standard deviation (SD)] population-weighted PM_1_, PM_2.5_, PM_10_, NO_2_, SO_2_, O_3_, and CO concentration levels were 45.1 (7.6) μg/m^3^, 58.5 (8.9) μg/m^3^, 79.5 (10.0) μg/m^3^, 23.9 (3.0) μg/m^3^, 13.9 (2.5) μg/m^3^, 83.6 (3.5) μg/m^3^, and 0.95 (0.07) mg/m^3^, respectively, and the total ischemic and hemorrhagic stroke cases were 44,043 and 9,434, respectively (Table 1).

**Table 1:** Means and standard deviations (SD) for air pollutant concentration levels and counts of stroke cases for the four phases.

**Phase**	**PM_**1**_**	**PM_**2.5**_**	**PM_**10**_**	**NO_**2**_**	**SO_**2**_**	**O_**3**_**	**CO**	**Counts of ischemic stroke cases**	**Counts of hemorrhagic stroke cases**
Lockdown phase in 2020	45.1 ± 7.6	58.5 ± 8.9	79.5 ± 10.0	23.9 ± 3.0	13.9 ± 2.5	83.6 ± 3.5	0.95 ± 0.07	44,043	9,434
Baseline phase	65.0 ± 14.2	90.4 ± 17.4	123.4 ± 20.0	51.1 ± 7.3	19.1 ± 4.1	47.2 ± 3.7	1.42 ± 0.18	54,244	9,868
Counterfactual lockdown phase	47.5 ± 7.9	76.2 ± 11.7	125.3 ± 15.8	40.3 ± 4.9	23.9 ± 5.3	73.4 ± 4.0	1.20 ± 0.12	55,442	10,615
Counterfactual baseline phase	53.4 ± 10.1	91.6 ± 18.0	151.3 ± 25.6	56.0 ± 8.3	30.0 ± 6.8	45.3 ± 4.4	1.50 ± 0.16	52,163	10,091

*The unit for PM_1_, PM_2.5_, PM_10_, NO_2_, SO_2_, and O_3_ is μg/m^3^, whereas that for CO is mg/m^3^*.

**Figure 2:**
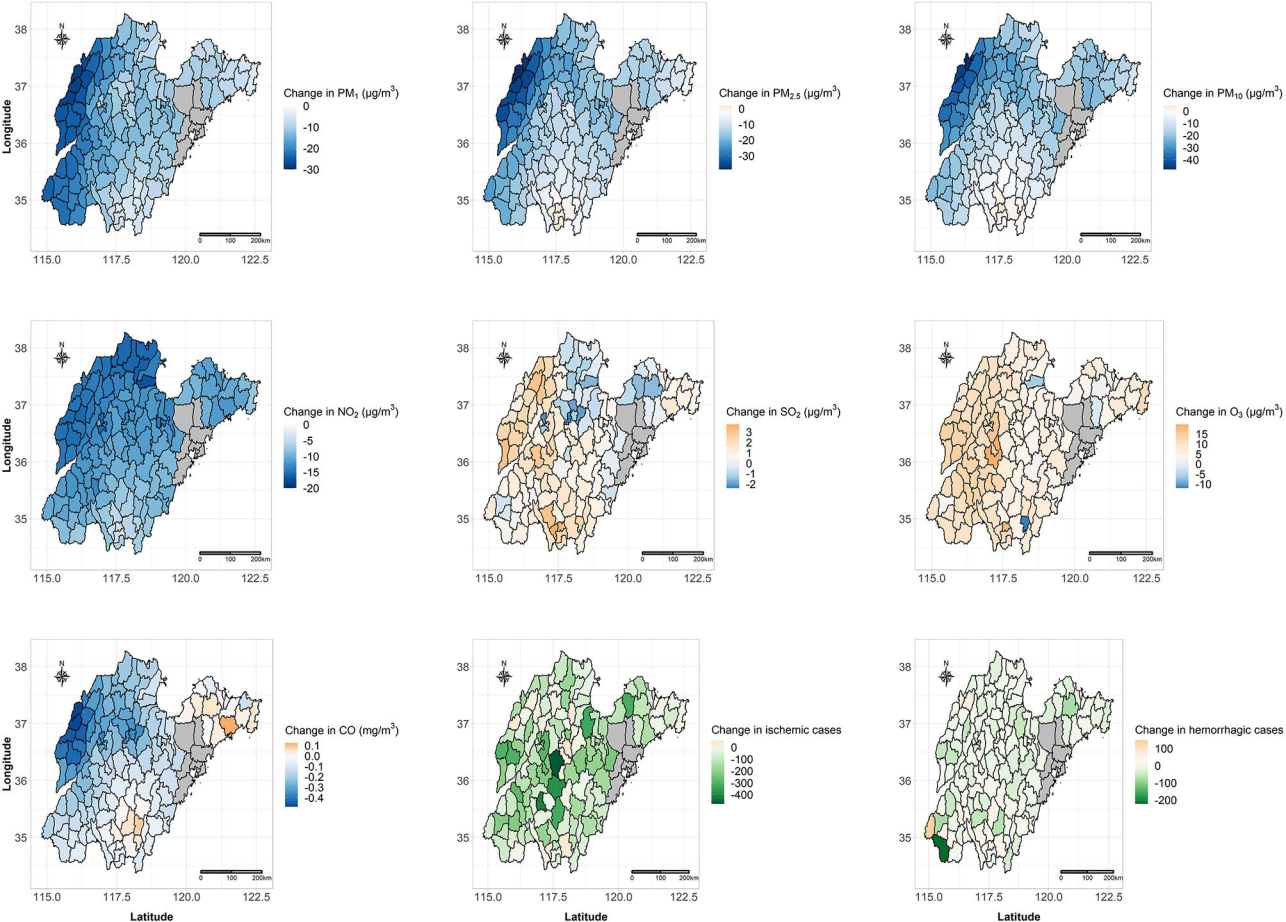
Changes in population-weighted average air pollutant levels and stroke cases between the lockdown phase in 2020 and the counterfactual lockdown phase of the past 2 years for each of the 126 included counties using the DID model.

Figure 2 shows the changes in population-weighted average air pollutant levels and stroke cases between the lockdown phase in 2020 and the counterfactual lockdown phase of the past 2 years for each county using the DID model. All 126 counties (100.0%) showed reductions in PM_1_ and NO_2_ levels, 122 counties (96.8%) showed reductions in PM_2.5_ level, 120 counties (95.2%) showed reductions in PM_10_ level, 110 counties (87.3%) showed reductions in CO levels, while 94 counties (74.6%) showed increases in SO_2_ levels, and 123 counties (97.6%) showed increases in O_3_ levels. The average changes in the population-weighted PM_1_, PM_2.5_, PM_10_, NO_2_, O_3_, and CO levels across the 126 counties were statistically significant (Table 2), and the respective estimates were −14.3 (95% confidence interval [*CI*]: −17.9, −10.8) μg/m^3^, −15.9 (95% *CI*: −20.5, −11.4) μg/m^3^, −16.9 (95% *CI*: −22.8, −11.0) μg/m^3^, −11.1 (95% *CI*: −13.1, −9.0) μg/m^3^, 8.4 (95% *CI*: 7.0, 9.9) mg/m^3^, and −0.16 (95% *CI*: −0.20, −0.11) μg/m^3^, representing a change of −30.2% (95% *CI*: −37.6%, −22.8%), −20.9% (95% *CI*: −26.9%, −15.0%), −13.5% (95% *CI*: −18.2%, −8.8%), −46.3% (95% *CI*: −54.7%, −37.8%), 11.5% (95% *CI*: 9.5%, 13.4%), and −13.1% (95% *CI*: −17.0%, −9.3%) relative to the counterfactual lockdown phase of the past 2 years, respectively. The average change in the SO_2_ level was 0.9 (95% *CI*: −1.0, 2.9) μg/m^3^, corresponding to a change of 2.3% (95% *CI*: −2.5%, 7.1%), which was not statistically significant.

**Table 2:** Changes in average air pollutant concentration levels and total counts of stroke cases in the lockdown phase relative to the counterfactual lockdown phase of the past 2 years.

**Outcome**	**Change in the lockdown phase**	**Proportion (%)**	***P*** **-value**
**Air pollutants**			
PM_1_ (μg/m^3^)	−14.3 (−17.9, −10.8)	−30.2 (−37.6, −22.8)	<0.001
PM_2.5_ (μg/m^3^)	−15.9 (−20.5, −11.4)	−20.9 (−26.9, −15.0)	<0.001
PM_10_ (μg/m^3^)	−16.9 (−22.8, −11.0)	−13.5 (−18.2, −8.8)	<0.001
SO_2_ (μg/m^3^)	0.9 (−1.0, 2.9)	2.3 (−2.5, 7.1)	0.357
NO_2_ (μg/m^3^)	−11.1 (−13.1, −9.0)	−46.3 (−54.7, −37.8)	<0.001
O_3_ (μg/m^3^)	8.4 (7.0, 9.9)	11.5 (9.5, 13.4)	<0.001
CO (mg/m^3^)	−0.16 (−0.20, −0.11)	−13.1 (−17.0, −9.3)	<0.001
**Ischemic stroke**			
Total	−15,315 (−27,689, −2,942)	−27.6 (−49.9, −5.3)	0.015
Age group			
18–64 years	−5,782 (−10,362, −1,203)	−29.0 (−51.9, −6.0)	0.013
65–74 years	−5,283 (−9,458, −1,108)	−28.4 (−50.8, −6.0)	0.013
≥75 years	−4,250 (−8,411, −90)	−25.2 (−49.8, −0.5)	0.045
Sex			
Male	−7,740 (−14,196, −1,285)	−26.0 (−47.7, −4.3)	0.019
Female	−7575 (−13,496, −1,654)	−29.5 (−52.5, −6.4)	0.012
**Hemorrhagic stroke**			
Total	−1,265 (−4,778, 2,249)	−11.9 (−45.0, 21.2)	0.490
Age group			
18–64 years	−744 (−1,857, 370)	−16.7 (−41.8, 8.3)	0.192
65–74 years	−259 (−1,586, 1,068)	−8.7 (−53.0, 35.7)	0.715
≥75 years	−262 (−1,529, 1,005)	−8.2 (−48.1, 31.6)	0.698
Sex			
Male	−520 (−2,208, 1,168)	−8.8 (−37.6, 19.9)	0.558
Female	−745 (−2,519, 1,029)	−15.7 (−53.2, 21.7)	0.418

*Ninety-five percent confidence intervals are presented in parentheses*.

In total, 110 counties (87.3%) showed a reduction in ischemic stroke cases, whereas 80 counties (63.5%) showed a reduction in hemorrhagic stroke cases. The population-weighted ischemic stroke cases were significantly reduced across the 126 counties (−15,315, 95% *CI*: −27,689, −2,942), representing a 27.6% reduction (95% *CI*: −49.9%, −5.3%) compared with that of the counterfactual lockdown phase. Furthermore, the reductions in the population-weighted ischemic stroke cases in both male and female subgroups, and all three age subgroups (18–64, 65–74, and ≥75 years) were also statistically significant. The total change in the hemorrhagic stroke cases was −1,265 (95% *CI*: −4,778, 2,249), which was not statistically significant. In addition, the changes in the hemorrhagic stroke cases were also not statistically significant in the sex or age subgroups. The sensitivity analysis revealed similar results, showing that the sex- and age-adjusted incidence rate for ischemic stroke decreased by 144 per 100,000 (95% *CI*: −257, −30, *p* = 0.013), and the sex- and age-adjusted incidence rate for hemorrhagic stroke increased by 5 per 100,000 (95% *CI*: −22, 32, *P*=0.730) during the lockdown for each county.

Since there were no statistically significant changes in the SO_2_ level and hemorrhagic stroke cases, we estimated the changes in the ischemic stroke cases attributed to the changes in the PM_1_, PM_2.5_, PM_10_, NO_2_, CO, and O_3_ levels across the 126 counties using the concentration–response function from previous studies (Table 3). The total avoided PM_1_-, PM_2.5_-, PM_10_-, NO_2_-, and CO–related ischemic stroke cases were 739 (95% *CI*: 641, 833), 509 (95% *CI*: 440, 575), 355 (95% *CI*: 304, 405), 1,132 (95% *CI*: 1,024, 1,240), and 289 (95% *CI*: 236, 340), respectively. The corresponding percentages of the avoided ischemic stroke cases were 4.8% (95% *CI*: 4.2%, 5.4%), 3.3% (95% *CI*: 2.9%, 3.8%), 2.3% (95% *CI*: 2.0%, 2.6%), 7.4% (95% *CI*: 6.7%, 8.1%), and 1.9% (95% *CI*: 1.5%, 2.2%), respectively. The increased O_3_ concentration levels across the 126 counties during the lockdown phase led to the increased counts of O_3_-related ischemic stroke cases at 48 (95% *CI*: 41, 54), corresponding to an increase of 0.3% (95% *CI*: 0.3%, 0.4%).

**Table 3:** Estimated counts of ischemic stroke cases related to the change in air pollutants in the lockdown phase relative to the counterfactual lockdown phase of the past 2 years.

**Air pollutants**	**Counts related to change in air pollutants**	**Percentage (%)**
PM_1_ (μg/m^3^)	−739 (−833, −641)	−4.8 (−5.4, −4.2)
PM_2.5_ (μg/m^3^)	−509 (−575, −440)	−3.3 (−3.8, −2.9)
PM_10_ (μg/m^3^)	−355 (−405, −304)	−2.3 (−2.6, −2.0)
NO_2_ (μg/m^3^)	−1,132 (−1,240, −1,024)	−7.4 (−8.1, −6.7)
CO (mg/m^3^)	−289 (−340, −236)	−1.9 (−2.2, −1.5)
O_3_ (μg/m^3^)	48 (41, 54)	0.3 (0.3, 0.4)

## Discussion

With the spread of COVID-19 worldwide, varying degrees of lockdown policies were implemented by the government to control this pandemic in most countries, and almost all aspects of life were affected during this period (2). The government of Shandong province imposed strict restrictions to lower the intensity of population outdoor activities (e.g., industry, traffic, construction, and entertainment), and the whole province was thrown into an unprecedented state of shutdown for about 6 weeks. This pandemic allowed us to estimate the changes in air pollutants and stroke cases relative to those during the same period in previous years using a quasi-experiment design.

The DID model indicated that if no lockdown occurred, the average concentrations of PM_1_, PM_2.5_, PM_10_, NO_2_, and CO across the 126 counties would increase by 30.2, 20.9, 13.5, 46.3, and 13.1%, respectively, whereas the average concentration of O_3_ would decrease by 11.5%. Most previous studies focused on the changes in commonly monitored air pollutants, and no study has quantified the change in PM_1_ levels during the lockdown in China (21–25). Our findings added to the growing literature evaluating the role of the COVID-19 lockdowns on ambient particulate matter, as we found substantial reductions in PM_1_, PM_2.5_, and PM_10_ levels during the COVID-19 lockdown phase. Moreover, the PM_1_ concentration level seemed to decrease more precipitously than those of PM_2.5_ and PM_10_. The detailed reason for the heterogeneity of the reduction effect of the lockdown on the particulate matter of different sizes is unclear and requires further exploration in future studies.

For other gaseous air pollutants, many studies from China and other countries (e.g., France, India, Italy, and Mexico) also found that the concentration of NO_2_ showed the most significant decrease and that of O_3_ showed an increase during the lockdowns (6, 12, 22, 23, 41, 42). A previous study based on 597 major cities worldwide reported similar findings (43). As nitrogen oxide is mainly related to emissions from motor vehicles, the remarkable reduction in the NO_2_ level may be due to the restriction of human traffic activities during the lockdowns (12, 42). Then, the reductions in the nitrogen oxide emissions because of the depressed anthropogenic activities may explain the observed increases in O_3_ during the lockdowns, as the drop in nitric oxide (NO) may slow down its interaction with O_3_ (NO + O_3_ = NO_2_ + O_2_). Thus, the O_3_ concentration may increase (7, 42).

Our results indicated that the COVID-19 lockdown did not result in significant changes in SO_2_ concentrations, which was consistent with several previous studies in northern and eastern China (44, 45). However, some studies also suggested that the levels of SO_2_ significantly decreased during the lockdowns in other regions of China (3, 6, 11). A possible interpretation for the limited influence of the lockdown on the SO_2_ levels in this area is that the additional emissions from coal-burning for residential heating because of the people staying at home during the relatively cold season may counterbalance the reduction in other emissions, such as those from factories (44, 45).

Several studies suggested significant drops in mortality or hospital admissions during the COVID-19 lockdowns, but only limited studies used stroke-related events as the outcome of interest (17, 46–48). For instance, in France, a significant drop in hospitalization related to stroke was observed only in the area most affected by COVID-19 compared with those in previous years. In contrast, no significant change in hospitalization for stroke was observed in the least affected area (17). Another study collected data from a hospital in Spain and reported that relative to March 2019, the number of stroke admissions declined by about 23% in March 2020 (46). Similarly, Zhao et al. reported that hospital admissions for stroke reduced by ~40% in February 2020 compared with that in the same period in 2019 in 227 hospitals across China (47). Kansagra et al. used the number of patients with stroke who underwent imaging as a surrogate for the number of cases of acute ischemic stroke in more than 800 hospitals in the United States, and they found that the number decreased by 39% during the early days of the pandemic (48). In this study, we estimated that the lockdown resulted in a drop of 27.6% in ischemic stroke cases compared with average values for the same periods of the previous 2 years. The reductions in PM_1_, PM_2.5_, PM_10_, NO_2_, and CO concentrations could have prevented as many as 4.8, 3.3, 2.3, 7.4, and 1.9% of the total ischemic stroke cases, respectively. The count of hemorrhagic stroke cases fell by a much smaller number (*n* = −1,265), which was not statistically significant. This finding is partly in line with previous studies indicating that exposure to air pollutants may exclusively increase ischemic stroke risks and not those of hemorrhagic stroke (20).

We noticed that not all avoided ischemic stroke cases could be attributed to the improved air quality during the COVID-19 lockdowns. Several other potential reasons may contribute to the observed decrease in the counts of ischemic stroke cases. First, the chance of family members and friends recognizing that a patient was having stroke symptoms may have been decreased because of the increased social isolation due to the lockdown (47). Second, most hospitals canceled their courses on stroke awareness education because of the imposition of social distancing (47). Third, patients with suspected acute stroke may have been worried about being infected with COVID-19 at hospitals (17). Fourth, some patients with severe stroke might have died at home (17). Fifth, evidence from other countries suggests that the reduced capacity of emergency services due to the burden of patients with COVID-19 may have limited the number of patients with stroke seeking essential medical services (49). However, only a total of no more than 800 COVID-19 cases were diagnosed across Shandong province during the lockdown, and this has limited influence on hospitals providing medical services for patients with stroke (50). Moreover, a recent study revealed that the onset-to-door time became even shorter during the lockdowns in Beijing, China, which might have benefited from the better traffic situation (51).

Our results imply that substantial health benefits could be achieved if stringent and effective control measures are implemented to tackle air pollution. Lockdowns are not appropriate for improving air quality in the long run, and persistent efforts are needed to reduce air pollution through a series of abatement measures for anthropogenic emissions (52). For example, policymakers could consider comprehensive strategies to upgrade local power and steel industries, provide more subsidies for public or electric transportation, and encourage households to transition from coal to cleaner energy sources (such as gas and electricity) (4).

Our study has several limitations. First, this is a quasi-experiment based on stroke count data at the population level. Thus, confounding factors at the individual level (e.g., tobacco smoking, hypertension, physical activity, and diet) (19, 53) could not be fully excluded, although the reductions in ischemic stroke cases were significant in all sex and age subgroups. However, we expect that the individual changes in the confounding factors did not cause substantial bias in the relation between the lockdown and stroke incidence at a population level (52). Second, the avoided ischemic stroke cases attributed to the changes in the air pollutant concentration levels were estimated using single-pollutant models. The strong correlation between the included air pollutants and the absence of epidemiological dose-response functions that account for the full suite of pollutants did not allow us to estimate the independent effect of each air pollutant. Therefore, some avoided stroke cases might have been counted more than once (52). Third, the method we used could not rule out the influence of meteorological conditions (e.g., air pressure, temperature, and wind field) on air pollutant concentration levels and the effect of extreme cold temperature on ischemic stroke incidence (54). However, a previous study suggested that the adjustment of meteorological variables had little effect on the estimated changes in air pollutant concentration levels during the lockdown in China (7). In addition, all people had to stay at home during the lockdown. Thus, we anticipate that the outdoor extreme cold temperature had a limited influence on the ischemic stroke incidence. Fourth, household air pollution (HAP) is also a risk factor for ischemic stroke (55), and the exposure level of HAP might be increased as most residents had to stay at home for longer durations during the lockdown (56). However, the additional counts of ischemic stroke resulting from HAP could not be estimated because of the lack of relevant data.

## Conclusion

The life-changing restrictions during the COVID-19 lockdown indirectly reduced the concentration levels of air pollutants (PM_1_, PM_2.5_, PM_10_, NO_2_, and CO) and subsequently reduced the associated ischemic stroke incidence. However, the health benefits brought by the lockdown are temporary, and long-term measures should be implemented to decrease air pollution levels and related health loss in the post-COVID-19 period.

## Data Availability Statement

The raw data supporting the conclusions of this article will be made available by the authors, without undue reservation.

## Ethics Statement

The studies involving human participants were reviewed and approved by Ethics Review Committee of Public Health, Shandong University. The Ethics Committee waived the requirement of written informed consent for participation.

## Author Contributions

HW: formal analysis, methodology, visualization, and writing the original draft. ZL and BZ: investigation, data curation, writing, reviewing, and editing. JW: methodology, resources, data curation, writing, reviewing, and editing. XL: validation, writing, reviewing, and editing. MZ: formal analysis, validation, and writing the original draft. WL: software, writing, reviewing, and editing. XG: supervision, resources, writing, reviewing, and editing. BX: conceptualization, supervision, funding acquisition, writing, reviewing, and editing. All authors contributed to the article and approved the submitted version.

## Funding

Funding was received from the National Important Project of the Ministry of Science and Technology in China (2017YFC1501404) and the Innovation Team of Climbing Program of Shandong University and the Youth Team of Humanistic and Social Science of Shandong University (20820IFYT1902).

## Conflict of Interest

The authors declare that the research was conducted in the absence of any commercial or financial relationships that could be construed as a potential conflict of interest.

## Publisher's Note

All claims expressed in this article are solely those of the authors and do not necessarily represent those of their affiliated organizations, or those of the publisher, the editors and the reviewers. Any product that may be evaluated in this article, or claim that may be made by its manufacturer, is not guaranteed or endorsed by the publisher.
