# Supplementary material for: Effects of the COVID-19 Lockdown on Air Pollutant Levels and Associated Reductions in Ischemic Stroke Incidence in Shandong Province, China
Source: Front Public Health. 2022 May 27;10:876615. doi: 10.3389/fpubh.2022.876615 (PMC9197688; doi:10.3389/fpubh.2022.876615)
Supplement: Supplementary file 1 [file Table_1.doc]

**Table S1.** Results of the parallel trend test for each of the air pollutants and stroke outcomes.

| Outcomes | Coefficient | Standard error | t | P value |
| --- | --- | --- | --- | --- |
| PM_1_ | 1.728 | 4.192 | 0.412 | 0.691 |
| PM_2.5_ | −5.750 | 9.100 | −0.632 | 0.545 |
| PM_10_ | −10.434 | 12.007 | −0.869 | 0.410 |
| NO_2_ | −0.133 | 1.371 | −0.097 | 0.925 |
| SO_2_ | −1.240 | 2.810 | −0.441 | 0.671 |
| CO | 0.051 | 1.860 | 0.027 | 0.979 |
| O_3_ | −0.035 | 0.089 | −0.391 | 0.706 |
| Ischemic stroke | −0.675 | 0.533 | −1.266 | 0.241 |
| Hemorrhagic stroke | −0.047 | 0.172 | −0.272 | 0.792 |

Note: Models were accounted for county-level clustering.

**Table S2.** Concentration−-response functions showing the association between air pollutants and ischemic stroke adapted adopted from two previous studies.

| Air pollutant | Unit | Coefficient | Lower bound | Upper bound | Author | Journal |
| --- | --- | --- | --- | --- | --- | --- |
|  |  |  |  |  |  |  |
|  |  |  |  |  |  |  |
|  |  |  |  |  |  |  |
| PM_1_ | 1 μg/m^3^ | 0.0009 | 0.0003 | 0.0015 | Chen L, et al. | Environmental Pollution |
| PM_2.5_ | 1 μg/m^3^ | 0.0006 | 0.0001 | 0.0011 | Chen L, et al. | Environmental Pollution |
| PM_10_ | 1 μg/m^3^ | 0.0004 | 0.0001 | 0.0007 | Chen L, et al. | Environmental Pollution |
| NO_2_ | 1 μg/m^3^ | 0.0018 | 0.0014 | 0.0022 | Tian Y, et al. | PLOS Medicine |
| CO | 1 mg/m^3^ | 0.0032 | 0.0020 | 0.0043 | Tian Y, et al. | PLOS Medicine |
| O_3_ | 1 μg/m^3^ | 0.0001 | -0.0001 | 0.0002 | Tian Y, et al. | PLOS Medicine |

**Reference**

Chen L, et al. Short-term effect of PM_1_ on hospital admission for ischemic stroke: A multi-city case-crossover study in China. Environmental Pollution. 2020, 260: 113776.

Tian Y, et al. Association between ambient air pollution and daily hospital admissions for ischemic stroke: A nationwide time-series analysis. PLOS Medicine. 2018, 15: e1002668.
